# Dural sac area is a more sensitive parameter for evaluating lumbar spinal stenosis than spinal canal area: A retrospective study

**DOI:** 10.1097/MD.0000000000009087

**Published:** 2017-12-08

**Authors:** Young Su Lim, Jong-Uk Mun, Mi Sook Seo, Bo-Hyun Sang, Yun-Sic Bang, Keum Nae Kang, Jin Woo Koh, Young Uk Kim

**Affiliations:** a Department of Anesthesiology and Pain Medicine, Institute for Integrative Medicine, Catholic Kwandong University of Korea College of Medicine, International St. Mary's Hospital, Incheon; b Department of Orthopaedic Surgery, Changwon Gyeongsang National University Hospital, Republic of Korea; c Department of Anesthesiology and Pain Medicine, National Police Hospital, Seoul, Korea.

**Keywords:** dural sac cross-sectional area, lumbar central canal spinal stenosis, spinal canal cross-sectional area

## Abstract

Narrowing of the dural sac cross-sectional area (DSCSA) and spinal canal cross-sectional area (SCCSA) have been considered major causes of lumbar central canal spinal stenosis (LCCSS). DSCSA and SCCSA were previously correlated with subjective walking distance before claudication occurs, aging, and disc degeneration. DSCSA and SCCSA have been ideal morphological parameters for evaluating LCCSS. However, the comparative value of these parameters is unknown and no studies have evaluated the clinical optimal cut-off values of DSCSA and SCCSA. This study assessed which parameter is more sensitive.

Both DSCSA and SCCSA samples were collected from 135 patients with LCCSS, and from 130 control subjects who underwent lumbar magnetic resonance imaging (MRI) as part of a medical examination. Axial T2-weighted MRI scans were acquired at the level of facet joint from each subject. DSCSA and SCCSA were measured at the L4-L5 intervertebral level on MRI using a picture archiving and communications system.

The average DSCSA value was 151.67 ± 53.59 mm^2^ in the control group and 80.04 ± 35.36 mm^2^ in the LCCSS group. The corresponding average SCCSA values were 199.95 ± 60.96 and 119.17 ± 49.41 mm^2^. LCCSS patients had significantly lower DSCSA and SCCSA (both *P* < .001). Regarding the validity of both DSCSA and SCCSA as predictors of LCCSS, Receiver operating characteristic curve analysis revealed an optimal cut-off value for DSCSA of 111.09 mm^2^, with 80.0% sensitivity, 80.8% specificity, and an area under the curve (AUC) of 0.87 (95% confidence interval, 0.83–0.92). The best cut off-point of SCCSA was 147.12 mm^2^, with 74.8% sensitivity, 78.5% specificity, and AUC of 0.85 (95% confidence interval, 0.81–0.89).

DSCSA and SCCSA were both significantly associated with LCCSS, with DSCSA being a more sensitive measurement parameter. Thus, to evaluate LCCSS patients, pain specialists should more carefully investigate the DSCSA than SCCSA.

## 1 Introduction

Lumbar central canal spinal stenosis (LCCSS) is a multifactorial pathological disorder of the concentrically narrowed spinal canal.^[1–4]^ It is the most common cause of disability in elderly and middle-aged patients.^[5,6]^ LCCSS typically causes bilateral or unilateral buttock pain, neurogenic intermittent claudication, and lower extremity heaviness, numbness, weakness, and pain.^[3,7]^ LCCSS can be defined as a change in the shape of the dural sac and central canal to triangles or flattened ovals ^[6,8]^, the spinal canal narrowing caused by arthrosis of the facet joint, mechanical lumbar nerve root compression, and obliteration of cerebrospinal fluid on axial images. ^[3,9]^

Magnetic resonance imaging (MRI) is most commonly used for the clinical assessment of degenerative LCCSS. LCCSS is a quantitative diagnosis that is made when the measurement of an individual is outside the range of normal. Thus, the criteria for LCCSS should be compared from an analysis of a normative distribution of measurements.^[10,11]^ In evaluating the severity LCCSS, dural sac cross-sectional area (DSCSA) and spinal canal cross-sectional area (SCCSA) are frequently measured by axial MRI.^[12–18]^ Previous studies have indicated that smaller minimum DSCSA is directly associated with more back and leg pain, shorter walking distances before claudication, and lower health-related quality of life.^[16,19–21]^ SCCSA is also related to subjective walking distance before claudication.^[22]^

DSCSA and SCCSA have been ideal morphological parameters for evaluating LCCSS. However, these parameters have not been compared. Moreover, no studies have evaluated the clinical optimal cut-off values of DSCSA and SCCSA. This study compared DSCSA and SCCSA between LCCSS patients and normal controls using MRI to determine which is more sensitive.

## 2 Materials and methods

### 2.1 Patients

We reviewed the medical records of patients who visited international St. Mary's pain clinic from March 2014 to January 2017 and who were diagnosed with LCCSS. The inclusion criteria were: baseline clinical manifestations compatible with LCCSS, such as leg or low back pain while walking; ≥ 60 years of age; MR images taken within 12 months of the last visit and available for review; and L4-L5 location for the most stenotic level. We excluded patients: previous lumbar surgery; past history of inflammatory disorders of the spine; previous spinal interventions, such as vertebroplasty or kyphoplasty; any congenital spine defect or disorder; metastasis of primary malignant disease in the lumbar spine; and peripheral arterial disease. A total of 135 patients enrolled after the diagnosis of LCCSS were confirmed by an experienced board-certified neuro-radiologist. To compare the DSCSA and SCCSA between patients with and without LCCSS, we also enrolled a control group of subjects who underwent lumbar MRI as part of a routine medical examination from March 2014 to January 2017. We only enrolled patients in the control group who did not have LCCSS-related symptoms. The DSCSA and SCCSA in the normal group were examined at the L4-L5 level as well. This study was registered at the University of Catholic Kwandong, Republic of Korea, Incheon (IS17RASI0008). The Institutional Review Board reviewed and approved the research protocol.

### 2.2 Imaging parameters

MRI was performed on an Avanto MR unit (Erlangen, Siemens, Germany) and 3 T Ingina (Achieva; Philips Healthcare, Best, The Netherlands) scanners. For lumbar MRI examinations, all axial, sagittal, and coronal T2-weighted images were obtained with a slice thickness <3 mm, 0.4-mm intersection gap, 3000 ms/90 ms repetition time/echo time, 30-cm field of view, 448 × 314 matrix, and >15 echo train length. Image parameters included slice thickness of 2 mm; intersection gap, 0.9 mm; repetition time/echo time, 2700 ms/95 ms; field of view, 30 cm; matrix, 358 × 512; and echo train length, 61. Nonenhanced T2-weighted sagittal images slice thickness, 3 mm; intersection gap, 0.4 mm; repetition time/echo time, 3625 ms/120 ms; field of view, 30 cm; matrix, 358 × 512; and echo train length, 16. All MRI data were transferred from the MRI unit to an INFINITT system (INFINITT Healthcare Co, Seoul, Korea).

### 2.3 Image analysis

**Figure 1:**
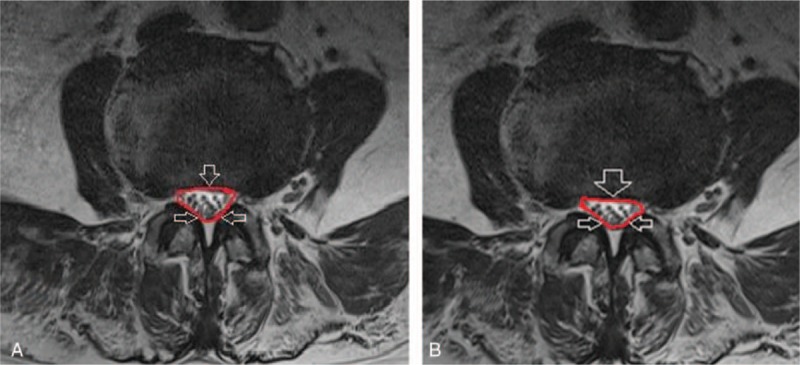
Measurement of dural sac cross-sectional area on MRI at the L4-5 level in the (A) control group and (B) lumbar central canal spinal stenosis group. MRI = magnetic resonance imaging.

**Figure 2:**
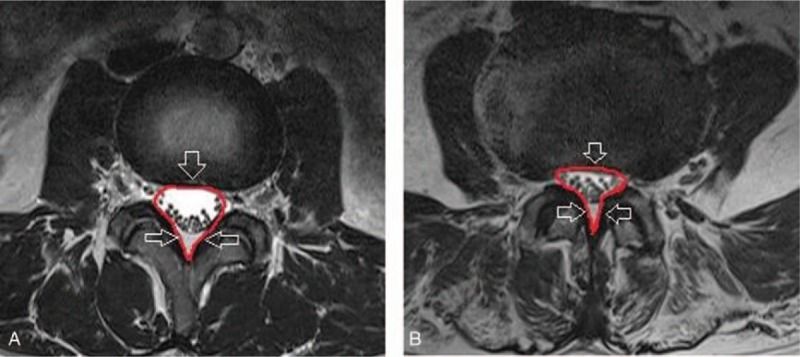
Measurement of spinal canal cross-sectional area on MRI at the L4-5 level in the (A) control group and (B) lumbar central canal spinal stenosis group. MRI = magnetic resonance imaging.

To obtain accurate morphologic results, MRI scans were magnified 2 times using the INFINITT system. Axial T2-weighted images were acquired at the level of facet joint for each subject. DSCSA and SCCSA were measured at the L4-L5 intervertebral level on the scans using a picture archiving and communications system. To determine DSCSA (Fig. 1) the dural sac was measured through the mid-point of the posterior border of the ligament flavum and disc on each side on axial T2 section of lumbar spine MRI scan.^[11]^ To determine SCCSA (Fig. 2) the posterior line of the disc was followed, turning down to reach the lumbar facet joint side on the opposite edge.^[22]^

### 2.4 Statistical analyses

Data are expressed as mean ± standard deviation (SD). Differences in demographic characteristics between the control and LCCSS groups were analyzed using unpaired *t* tests. The validity of the DSCSA and SCCSA for the diagnosis of disease was estimated by Receiver Operator Characteristics (ROC) curves, cut-off values, area under the curve (AUC), sensitivity, and specificity with 95% confidence intervals (CIs). AUC was calculated independently in the final results to demonstrate the additional value gained from the addition of each parameter. A *P* value <.05 was considered statistically significant. SPSS for Windows version 21 (IBM SPSS Inc, Chicago, IL) was used for the statistical analyses.

## 3 Results

The LCCSS group included 135 individuals comprising 80 (59.2%) men and 55 (40.8%) women with a mean age of 65.70 ± 10.08 years (range, 60–88 years) (Table 1). The control group included 130 people (47 males and 83 females) with a mean age of 65.33 ± 9.68 years (range, 60–86 years). There were no significant differences in the demographic characteristics between the 2 groups.

**Table 1:**
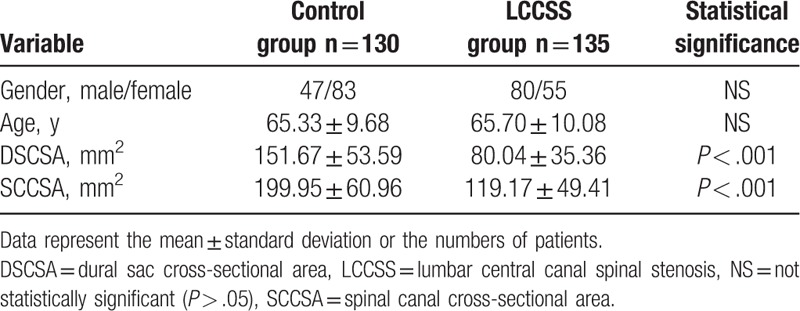
Comparison of the characteristics of control and LCCSS groups.

The average DSCSA was 151.67 ± 53.59 mm^2^ in the control group and 80.04 ± 35.36 mm^2^ in the LCCSS group. The average SCCSA was 199.95 ± 60.96 mm in the control group and 119.17 ± 49.41 mm^2^ in the LCCSS group. LCCSS patients had significantly lower DSCSA (*P* < .001) and SCCSA (*P* < .001) than controls (Table 1).

**Figure 3:**
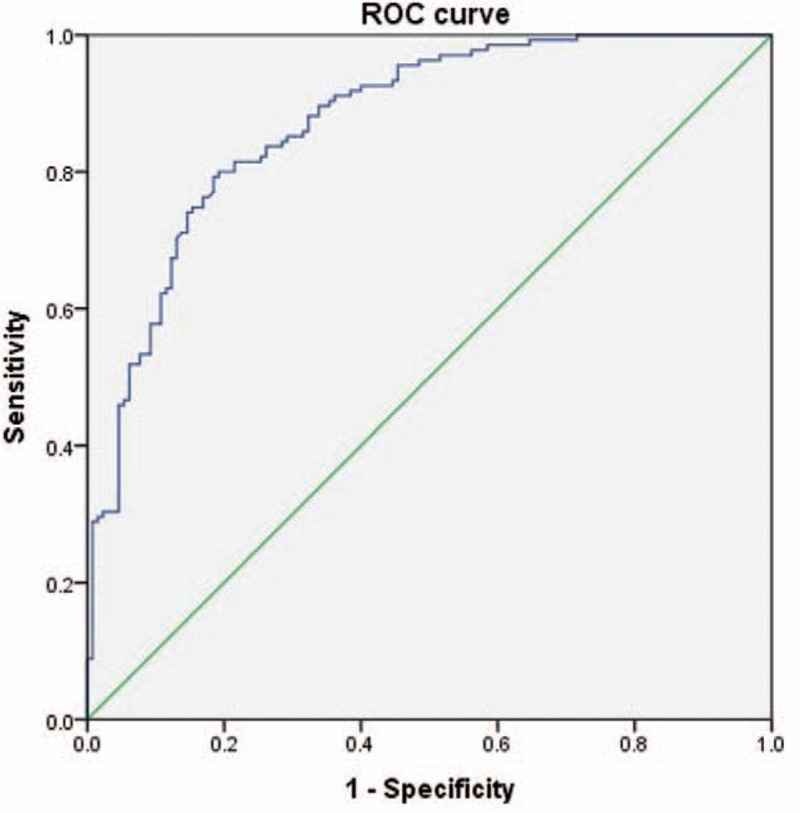
Receiver operating characteristic (ROC) curve of DSCSA for the prediction of LCCSS. The optimal cut-off point of DSCSA was 98.60 mm2 , with sensitivity 80.0%, specificity 80.8%, and area under the curve of 0.87 (95% CI = 0.83–0.92). AUC = area under the curve, CI = confidence intervals, DSCSA = dural sac cross-sectional area, LCCSS = lumbar central canal spinal stenosis.

**Figure 4:**
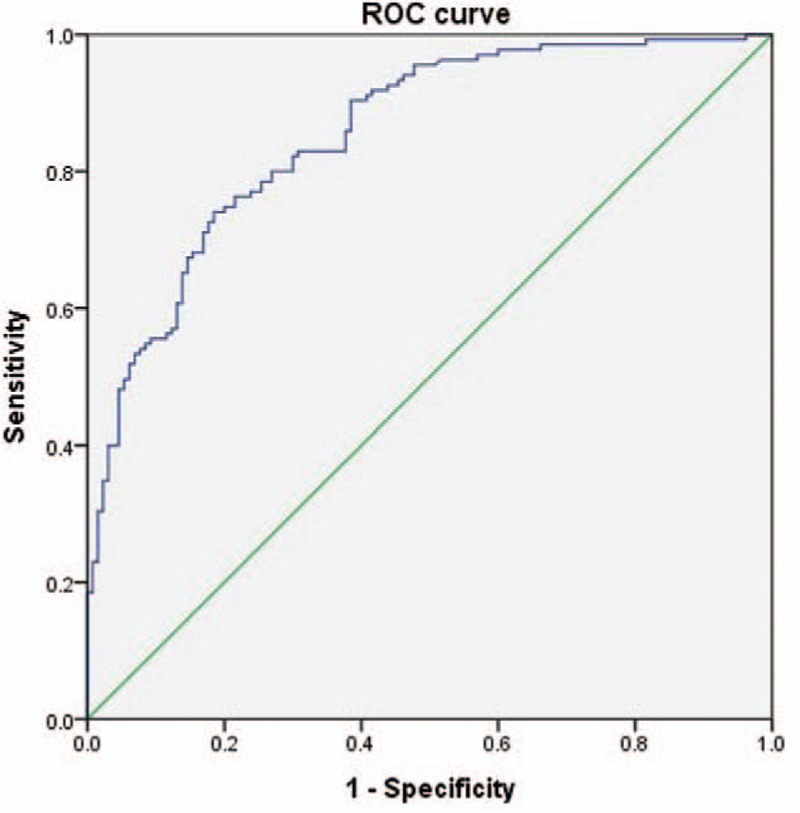
Receiver operating characteristic (ROC) curve of SCCSA for the prediction of LCCSS. The optimal cutoff point of SCCSA was 147.12 mm^2^, with sensitivity 74.8%, specificity 78.5%, and area under the curve of 0.85 (95% CI = 0.81–0.89). AUC = area under the curve, CI = confidence intervals, LCCSS = lumbar central canal spinal stenosis, SCCSA = spinal canal cross-sectional area.

Regarding the validity of both DSCSA and SCCSA as predictors of LCCSS, ROC curve analysis revealed an optimal cut-off value for DSCSA of 111.09 mm^2^, with 80.0% sensitivity, 80.8% specificity, and AUC of 0.87 (95% CI, 0.83–0.92) (Table 2, Fig. 3). The best cut off-point of the SCCSA was 147.12 mm^2^, with 74.8% sensitivity, 78.5% specificity, and AUC of 0.85 (95% CI, 0.81–0.89) (Table 3, Fig. 4).

**Table 2:**
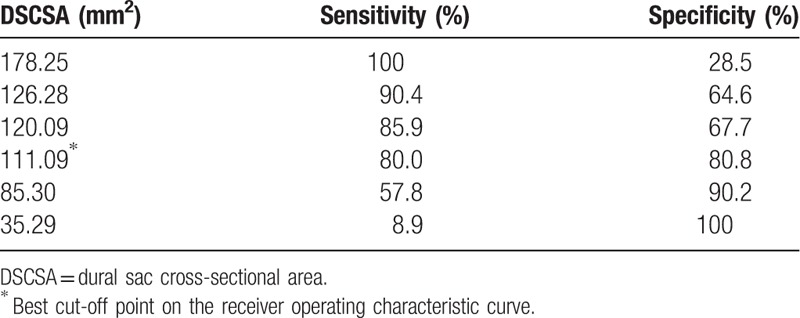
Sensitivity and specificity of each DSCSA cut-off.

**Table 3:**
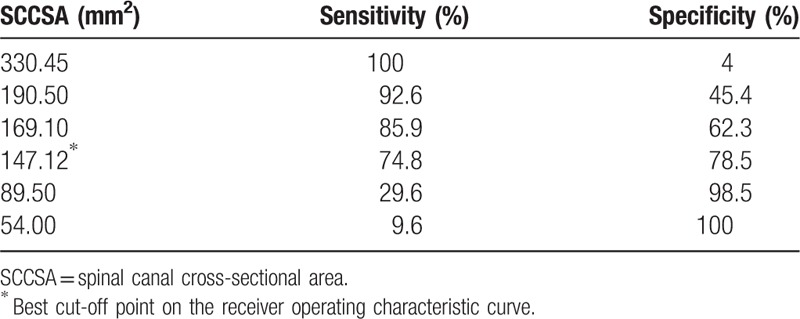
Sensitivity and specificity of each SCCSA cut-off.

## 4 Discussion

The most common spinal disorder in elderly patients is LCCSS. The consequences include intermittent neurogenic claudication and low back pain.^[3,5,10]^ LCCSS results from a decrease in the antero-posterior, transversal, or combined diameter secondary to hypertrophy of the facet joints, loss of disc height with or without herniation of the intervertebral disc, and hypertrophy of the ligamentum flavum.^[11]^ The clinical diagnosis of LCCSS is currently based on clinical evaluation, history, and confirmatory imaging demonstrating central spinal canal narrowing.^[23,24]^ The most frequently applied criteria were measurement of the anterior-posterior diameter of the cross-sectional area of the dural sac and of the osseous spinal canal for LCCSS.^[24]^ Thus, the analysis of the DSCSA or SCCSA is very important to diagnose LCCSS.^[24,25]^

The purpose of this research was to analyze these 2 important morphological parameters to obtain which is more sensitive. To the best of our knowledge, this is the first comparison of the optimal cut-off values of DSCSA and SCCSA in affected individuals. DSCSA proved to be the more sensitive parameter for evaluating LCCSS than the SCCSA. This finding should immediately contribute to clinician‘s understanding of LCCSS.

Studies have evaluated the associations between the DSCSA on MRI and the symptoms of LCCSS.^[20,26]^ Smaller DSCSA was directly related to lower health-related quality of life, more back and leg pain, and shorter walking distances before claudication.^[16]^ DSCSA was proposed as the most specific and sensitive morphologic parameter predicting the absence or presence of leg pain.^[27]^ Another study demonstrated that the ratio between the DSCSA of the vertebral body can be used as a diagnostic marker to predict the occurrence of LCCSS.^[11]^ Narrow DSCSA was significantly associated with the presence of low back pain after adjustment for body mass index, age, and sex.^[28]^

Studies have also investigated the associations between SCCSA determined on MR images and the signs and symptoms of LCCSS. In 1 study, preoperative MRI measurements of SCCSA had value for the treatment selection of lumbar disc herniation.^[29]^ The finding of a statistically significant association between the SCCSA and walking distance provided evidence of an association between a larger SCCSA and a longer subjective walking distance before the onset of claudication.^[22]^ All these previous studies indicated that the diagnostic sensitivity of the 2 morphological parameters made them good indicators.

A DSCSA >70 mm^2^ was suggested to represent critical stenosis^[21]^, with a significant correlation with the Oswetry disability index noted.^[21,30]^ Presently, the optimal cut-off value of 111.09 mm^2^ for DSCSA had high sensitivity (80.0%) and specificity (80.8%) for predicting LCCSS. This optimal cut-off value is less than some prior studies, but is similar with the report of a cut-off <100 mm^2^ as being stenotic.^[13]^

Concerning SCCSA, the optimal cut-off value was 147.12 mm^2^, with 74.8% sensitivity, 78.5% specificity, and an AUC of 0.85 (95% CI, 0.81–0.89) to predict LCCSS. In the absence of any other reported cut-off value, this SCCSA value represents the current standard. We measured the DSCSA and SCCSA at the L4-5 level to obtain the most accurate measurements of thickness. Prior studies significantly correlated the L4-5 level of degenerative spondylolisthesis with the Oswetry disability physical function score.^[21,31]^ A positive correlation was reported between leg pain visual analog scale score and severity of stenosis at L4-5.^[21]^ Presently, we strictly controlled for age (all patients exceeded 60 years of age) in light of the observation that the morphologic parameters become thicker with age.^[7]^

There were several limitations to the current study. Anatomically, degenerative lumbar spinal stenosis can involve the central canal, foramina, or subarticular (lateral recess) location, or combination of these locations.^[32]^ However, we focused on LCCSS only. Second, there are different methods to investigate spinal stenosis, such as sedimentation sign and morphologic analysis, effectively discriminate spinal stenosis.^[33,34]^ However, we only measured DSCSA and SCCSA. Thus, our results could be limited. Third, the principal methodological limitation was the retrospective evaluation. Fourth, the research population included a small number of LCCSS patients. Baseline characteristics of the patient population such as body mass index, weight, height can vary widely. Fifth, this research did not analyze axial-loaded MR imaging. Evaluating the severity of spinal canal narrowing on axial-loaded MRI scans is more beneficial for accurate diagnosis that conventional MRI.^[15]^ Sixth, we only deals with DSCSA and SCCSA of L4-L5 level. We strongly suggest that future studies examine both DSCSA and SCCSA at the L5-S1 level. Lastly, the determination of LCCSS is not simply based on morphologic parameters.^[24]^

Despite these limitations, the results are important as this is the first trial to compare DSCSA and SCCSA.

## 5 Conclusions

Although the DSCSA and SCCSA were both significantly associated with LCCSS, DSCSA was a more sensitive measurement parameter for LCCSS than was SCCSA. We identified the best cut-off value of the DSCSA as 111.09 mm^2^, with 80.0% sensitivity and 80.8% specificity. The best cut-off value of the SCCSA was 147.12 mm, with 74.8% sensitivity and 78.5% specificity. When evaluating patients with LCCSS, physicians should carefully assess the DSCSA rather than the SCCSA.
